# The Commissioner's Summing-Up, and the Verdict

**Published:** 1852-04-01

**Authors:** 


### THE COMMISSIONER'S SUMMING-UP, AND THE VERDICT.

After the third interview with Mrs. Cumming on the previous evening, the jury assembled on the 24th January for the sixteenth day. The Foreman of the Jury expressed the opinion of the jury that it was not necessary to ask the learned Commissioner to sum up the evidence.

Another Juryman adverted to the circumstance that the learned Serjeant (Wilkins) had pointed out a distinction between the law of Scotland and England, and said it would be satisfactory to him if the Commissioner were to touch upon that subject.

The Commissioner said:—The case was now ripe for the determination of the jury, and they were to decide it upon their own opinion and not upon the opinion of anybody else. What might have been best for the personal comfort of this lady, and for the protection of her property, was not a question for them now to consider. They had only to consider upon the evidence which had been laid before them—what opinion they formed as to the existing state of her mind, and what it had been for some years past.

The most important evidence in almost all these cases was what passed between the jury and the patient. The Commissioner always liked the presence of counsel on these occasions; it was very difficult to avoid the appearance of putting questions with a view to one side or the other; indeed, it was self-evident, on looking through the short-hand writers' notes of the first examination, that he was putting questions which ultimately proved very irrelevant to the real issue. At that time they only knew one part of the case. The presence of counsel was desirable to see that the party should not be subjected to a kind of cross-examination which would be unfair. These things should be treated in a conversational tone, to get out the real merits and the real feeling and characters of the individuals.

The issue before the jury was this:—Whether she was now a lunatic, an idiot, or a person of unsound mind, so that she was incompetent to take care of herself and her property. If they came to that conclusion, they would have then to point out from what particular day they thought she had been in that state; and whether she had continued in that state from that particular date to the present time. Those who had taken out the Commission asked the jury to find that she was actually of unsound mind, and suggested that the jury should carry that back as far as May, 1846. The Commissioner, therefore, directed the attention of the jury to the evidence relating to that period.

It was not for the jury to consider whether this lady was or was not a fit subject for a lunatic asylum; all they had to consider was, what was and what had been the state of her mind. Neither was it the province of the jury to consider what the effect of their verdict might be upon other parties. Any transactions of an antecedent date to the verdict, they were to look at only so far as they bore upon the state of her mind. It was quite clear that in this particular case the character of individuals was, to a certain extent, indirectly concerned. They would abstain from expressing an opinion upon anything that any person might have done; and only take into consideration the proceedings of other parties as evidence to assist in forming their opinion.

The Commissioner then referred to the three legal phrases, "idiot," "lunatic," and "unsound mind," which he had explained on opening the Commission. He said, for a great many years there was some doubt whether persons of unsound mind in one sense—he meant with reference to the management of their property—persons of senile imbecility, could by possibility come under a Commission of this kind. It had been decided by Lord Eldon, over and over again, that if the jury were satisfied that there was imbecility from old age to such an extent as to deprive a person of the power of managing himself or his property, and if the jury were satisfied that the party was of unsound mind—if there were evidence sufficient for that—and if the jury were satisfied of that, then the case came within the terms of this Commission. It was not that imbecility was insanity—it was not that the want of power of managing property was insanity—but it was the fact when the acts and deeds proved before the jury led them to the conclusion that the person was of unsound mind. It was utterly impossible to define, with anything like accuracy, what was or was not unsoundness of mind. They must form their own opinion, in each particular case, from the facts which were laid before them. There was one observation he should make, in consequence of what had been said by the learned Serjeant as to whether it was sufficient to be satisfied that the party was not competent to manage his property. The answer was, that the person might be insufficient for the proper management of his property, and might have a partial loss of memory, and yet they might not be led to the conclusion that that person was of unsound mind; and, on the contrary, there might be sufficient evidence before them that there was such imbecility of mind as amounted to unsoundness of mind. The mind might be diseased to such an extent as to satisfy them, in making a return to an inquisition of this kind, that the person was of unsound mind.

It was laid down in Shelford, "In deciding whether a party is of sound or unsound mind, one of the most important points to be considered, and which should be distinctly ascertained as far as it can be fixed, is, what is the test and criterion of unsound mind, and where eccentricity or caprice ends and derangement commences. Derangement assumes a thousand different shapes as various as the shades of human nature. It shows itself in forms very dissimilar both in character and in degree. It exists in all imaginable varieties—from the frantic maniac chained down to the floor, to the person apparently rational on all subjects and in all transactions save one, and whose disorder, though latently perverting the mind, yet will not be called forth except under particular circumstances, and will show itself only occasionally." Then, in attempting to define sound and unsound mind, this gentleman said:—"A sound mind is one wholly free from delusion, and all the intellectual faculties existing in a certain degree of vigour and harmony; the propensities, affections, and passions being under the subordination of the judgment and the will: the former being the controlling power, with a just perception of the natural connexion or repugnancy of ideas. Weak minds, again, only differ from strong ones in the extent and power of their faculties; but unless they betray symptoms of a total loss of understanding, or of idiocy, or of delusion, they cannot properly be considered unsound."

Then he went on to define, as well as he could, an unsound mind:—"An unsound mind is marked by delusion, mingles ideas of imagination with those of reality; those of reflection with those of sensation; and mistakes the one for the other; and such delusion is often accompanied with an apparent insensibility to, or perversion of, those feelings which are peculiarly characteristic of our nature," &c., &c.

Then with reference to delusion:—"The true criterion, the true test of the absence or presence of insanity, where there is no frenzy or raving madness, seems to be the absence or presence of what, used in a certain sense of it, may be comprised in a single term—viz., delusion. Wherever the patient once conceives something extravagant to exist, which has still no existence whatever but in his own heated imagination; and wherever, at the same time, having once so conceived, he is incapable of being, or at least of being permanently, reasoned out of that conception—such a patient is said to be under a delusion. Insane delusion consists in the belief of facts which no rational person would have believed. This delusion may sometimes exist on one or two particular subjects; though, generally, there are other (symptoms), such as eccentricity, violence, suspicion, exaggeration, inconsistency, &c., which may tend to confirm the existence of delusion, and to establish its insane character."

The Commissioner then quoted some passages from Ray, to exemplify the difficulty of drawing definitions of insanity. The Commissioner then went on to observe:—In this case the medical men who had been brought before the jury did not agree in their opinion. Different men saw different facts, and came to different conclusions upon those facts; and it was only those who had seen the whole case, and who had seen the lady herself, who could form an accurate opinion as to the existing state of things.

Now, in consequence of what had been said as to whether incompetency to manage property would be a sufficient ground for finding Mrs. Cumming to be of unsound mind, the Commissioner would refer to what was laid down in the matter of Holmes, in the 4th vol. of *Russell's Reports*. In that case a commission of lunacy had issued against Mr. Holmes, and the jury found, "That the Rev. Holmes is not a lunatic, but that partly from paralysis, and partly from old age, his memory is so much impaired as to render him incompetent to the management of his affairs, and consequently of unsound mind; and that he has been so for the term of two years last past." An objection was taken to that finding, inasmuch as it was no answer to the inquiry which the jury were directed to make. It was said the latter clause ("consequently of unsound mind,") was an expression of an inference which the jury had drawn from the fact which they had previously found, and not a distinct and independent finding. This point came before the Lord Chancellor, who gave this opinion:—"It is not necessary to go back to old authorities. We have the law, as it is now to be administered, clearly expounded by Lord Eldon in Ridgway *v.* Darwin. Of late the question has not been whether the party is absolutely insane, but the Court has thought itself authorized (though certainly many difficult and delicate cases with regard to the liberty of the subject occur on that) to issue the commission, provided it is made out that the party is unable to act with any proper and provident management; liable to be robbed by any one; under that imbecility of mind, not strictly insanity, but as to the mischief, calling for as much protection as actual insanity." The law thus stated by Lord Eldon had been acted upon for years; the legislature had not thought proper to interpose. In the case cited the Lord Chancellor directed a fresh commission to issue; and the jury were satisfied as to the insanity of the individual.

That case, therefore, showed, that juries were apt to consider capability, or incapability, of managing property as evidence upon the soundness or unsoundness of mind of the party; and if they came to the conclusion that there was sufficient evidence to show them the party's mind was unsound, it was for them to say that; and not for the Chancellor.

The jury would therefore consider the evidence with reference to this lady's capacity to manage her property. A slight incapacity would certainly not justify them in pronouncing her of unsound mind, but a positive incapacity would justify them in coming to such a conclusion.

With reference to the Scotch law: in England there was no distinction between taking charge of the person and charge of the property. In Scotland the law was very different. There they had a power of appointing a curator of the property only, and the individual was at perfect liberty to do what he thought fit.

The Commissioner then alluded to the positive hatred of her children which Mrs. Cumming had manifested before the jury; her refusal to answer questions relating to her property. He should have liked those who had the conduct of her case to know the position she was in by refusing to answer. It was impossible not to draw inferences when a person will not or cannot answer. He should have been better satisfied if they could have had a better history of her property.

The Commissioner then adverted to the bearing of the different facts in evidence. It was clear that keeping cats was but an eccentricity at most, unless it was carried to a very great extent. In this case it was not carried to any very great extent. Another leading characteristic was her extreme feelings against her children. The jury were to determine whether those feelings were the feelings for offence given, or whether they were from any morbid disposition antecedent to those feelings. They would consider the marriage of Mrs. Hooper an undoubted act of disobedience; and what had taken place in 1846. Then there was the will, a transaction which could not fail to be matter of suspicion in the minds of the family. She herself assured them she had executed no will. (Mr. Southgate reminded the Commissioner that Mr. Joseph Haynes said it was never engrossed.) It could not be considered as a positive act of insanity. Another part of the case was her habit of changing her residences. It was said she had done that because she had been annoyed wherever she had been. There was also a peculiarity with reference to her solicitors. In 1844, Mr. Dangerfield was her solicitor. Mr. Haynes acted for her through the medium of his brother. Then she seemed to have discarded Mr. Haynes, and to have taken to Mr. Thorne, and then came back to Mr. Haynes.

A material part of the case was her conduct in 1846 when at the asylum. She came before the jury under the former commission. The jury were to assume what took place upon that occasion precisely as if there had been no inquisition at all. No verdict had been given; no opinion had been expressed. He (the Commissioner) had done that which he did not now regret; he had told her she was a free agent, but he had gone further; the lady appeared for the first part of the day without counsel or solicitor. Before, therefore, he discharged the jury, he had asked her whether she had confidence in her counsel and solicitor, who were then appearing for her, taking care not to ask her anything about the arrangement, because that was a thing to which he could not be a party; it had seemed to him that difficulties would arise if the parties were not willing to carry out that arrangement.

Then there was that which was always an important part of the case, but with reference to which they were here to a certain extent left without the usual assistance: he meant the medical testimony; they had a great deal of it, but it was of a conflicting character. On reading through the decision of Lord Campbell, in the House of Lords, in the case to which he had adverted the other day, it seemed to him quite clear, that if the jury were to ask medical men to give their opinion upon what had taken place in court, that would be putting the medical men in the position of the jury. They must have the opinion of a medical man in two points of view—what he says he knows, and the opinion he forms from that knowledge. He, therefore, could not allow the medical men to give the jury that kind of assistance which had been allowed heretofore. They would hereafter go through the evidence given by these medical men. The number examined seemed to be pretty nearly equal upon each side; but that must not guide them: they must look at how often each medical man had attended her, what opportunities he had had of observing her, and of obtaining information, and at the grounds which he gave for the opinions he formed.

It did not seem to him that what the effect was of the settlement of this lady's property had been accurately stated on either side. It might not, indeed, bear materially upon the case, except as to the impression which this lady had as to the extent of her property in 1846—he meant, whether she was entitled to it for life or not. If she held for life only, then the daughters would be giving up to her one-third; if, on the other hand, she had an absolute interest, then she would be giving up two-thirds; having, in either case, the right of disposing of one-third. The Commissioner then quoted the terms of the original settlement of the property, the subsequent settlement by her father in 1809, and his will; he gave his view of the legal position of the parties with reference to the property. It was difficult to say whether she had an estate absolutely, or for life. She had the option of saying, "I will not adopt the deed of 1809; I will take the whole property under the original settlement;" or, on the other hand, "I will not adopt the deed of 1809, and then I will take under the will;" and in the latter case she would have taken the personal property. The object of the suit of 1848, therefore, was, that she should say whether she would take under her father's will or not; and in the result, she gave up her interest under that will, which gave an absolute interest in the personal property of the father, Mr. Prichard, to the children. The Commissioner then pointed out to the jury, that they had to ascertain in what state of mind they thought this lady now was. In order to ascertain that, it was necessary that they should look back. If they should think that she was now of sound mind, there their duty would end. If they thought she was of unsound mind, then they would point out some particular day from which, in their opinion, she had been in that state.

The jury retired to consider their verdict; their deliberation lasted one hour and twenty-five minutes. On returning into court, the Foreman spoke as follows:—"The jury have taken the case into great consideration, and we come into court, sir, to give our verdict—That the jury are unanimously of opinion, that Catherine Cumming is of unsound mind, and incapable of managing herself and her property; and that she has been so from the first day of May, 1846."

The jury then signed the precept in accordance with their verdict, and the proceedings terminated.

